# Schwartz Value Clusters in Modern University Students

**DOI:** 10.3390/bs10030066

**Published:** 2020-03-08

**Authors:** Olga V. Maslova, Dmitry A. Shlyakhta, Mikhail S. Yanitskiy

**Affiliations:** 1 Social and Differential Psychology Department, Peoples’ Friendship University of Russia (RUDN University), 6 Miklukho-Maklaya Str., Moscow 117198, Russia; shlyakhta-da@rudn.ru; 2 Clinical Psychology Department, I.M. Sechenov First Moscow State Medical University (Sechenov University), 8 Trubetskaya Str., Bldg. 2, Moscow 119991, Russia; 3 Social Psychological Institute, Kemerovo State University, 6 Krasnaya Str., Kemerovo 650000, Russia; direktorspi@kemsu.ru

**Keywords:** personal values, values hierarchy, values clusters

## Abstract

People differ in their value hierarchies, i.e., in the importance they attach to basic personal values. A large number of studies were performed to establish similarities and differences between national, ethnic, or professional groups in terms of Schwartz’s values structure. In addition to this sample-level approach, we found it useful to disclose a number of subgroups within those larger social groups, which are more homogeneous in themselves and reflect the individual-level types of personal values systems. The study was performed on university students (n = 1237) who were asked to fill in the SVS и PVQ Schwartz’s questionnaires. The sample was then treated with the K-means cluster analysis, which resulted in the division of the initial sample into three subgroups or clusters according to their values hierarchy being measured separately at the (1) Normative Ideals scale and (2) the scale of Behavioral Priorities. These clusters were equally common among male and female students, but they were unequally found in young people coming from different ethnic groups and regions, demonstrating the role of socio-cultural environment in building up personal values. The results may extend our capabilities for the prediction of the social, economic, and political behavior of the younger generation.

## 1. Introduction

Values are broad desirable goals that define the way people select actions, evaluate other people and events, and explain their behavior and judgment [1,2,3]. Individuals are likely to differ in their value hierarchies, which reflect both societal influences and personal experiences. Values as guiding principles in the life of a separate person as well as of any kind of social entity constitute a very important part of cultural and cross-cultural research. They determine what is really important, they guide people’s behavior and reflect real differences between cultures, social classes, occupations, religions, and political orientations [2,3,4]. Most cross-cultural researchers consider values to be a major cause of differences across people and societies. While being stable enough through the course of one’s life, values may change according to changes in economic development or personal life events [5,6,7]. Several researchers have proposed various grounds and ways of constructing the typology of values, in some cases determining the selection of certain value types [8,9,10]. R. Inglehart describes the so-called “materialists” and “post-materialists” who focus on different systems of values [8]. 

Shalom H. Schwartz made an important contribution when he noted the importance of understanding values as a system, rather than concentrating on individual values [4,11]. He proposed a circular structure that explains the interrelationships between his 10 basic values, based on their motivational conflicts and compatibilities. This structure has been confirmed in many studies fulfilled in more than 80 countries which proved that these basic values are universal for many countries and cultures [3,12,13]. In his works, Schwartz and his colleagues offer a profound theoretical approach to perform values-based studies within and across countries.

However, most cross-cultural studies that examine values systems survey large and rather heterogeneous groups (e.g., national or ethnic groupings), with a subsequent analysis of means or ranking of the studied values. In spite of their acknowledgment that individuals may differ in their value priorities, they seldom tend to study this difference itself. Meanwhile, such sample-level hierarchies may hide important information about differences in values systems; these differences may be “lost” or simply smoothed away [6]. It would be useful to examine more homogeneous subgroups within the large groups or societies according to the personal value hierarchies which may substantially differ in these subgroups. Most comparative values studies should distinguish subgroups in their subjects based on such characteristics as gender or generation, e.g. [14,15]. Perhaps homogeneous subgroups may be found apart from these major divisions. This finding would result in a new kind of grouping that reflect the individual-level compatibilities and conflicts inherent within Schwartz’s values structure. 

Surprisingly, only a few studies propose the distinction of subgroups according to personal value hierarchies. For example, M.K. Kozan found support for values-based subgroups that reflected Schwartz’s [3,4] model, which he termed *traditionals* who emphasized conservative values; *power seekers,* who emphasized power and hedonism; *egalitarians* who emphasized self-transcendence values; and *stimulation seekers* who emphasized openness-to-change values [16]. One more profound study with the use of cluster analysis was fulfilled by J. A. Lee and colleagues [6]. In their studies, performed on international travelers from the USA and from China (Study 1) and young people belonging to the very different cultures of the USA and China (Study 2), similar subgroups or clusters were established. Four similar values-based clusters—*self-enhancement*, *openness to change*, *in-group* and *universalism*—were found in two matched samples from two very different cultures. In each case, the clusters reflected the shared and conflicting motivations described by Schwartz [3], as neighboring values with shared motivations were similarly important while conflicting motivations were similarly unimportant within each cluster. 

From the just listed studies, one may see that a values-based cluster analysis offers the potential to better understand the range of value hierarchies that exist both within and between different cultures and countries.

The main purpose of this study was to distinguish the general types of value hierarchies existing among contemporary university students with the use of the above-mentioned approach, as well as examine the supposed differences between genders, geographical regions, and ethnic groups.

## 2. Materials and Methods

The sample included 1237 university students aged 16 to 25, mean age 19.4 (SD = 2.09), 33 percent being male and 67 percent female. By ethnic identity, the sample included 880 Russians, 139 Buryats, 125 Ukrainians, and 93 representatives of other ethnic groups, either of minor indigenous groups or migrants from bordering countries. Depending on the site of residence, each respondent was assigned to one of the following regions: Siberia (cities of Kemerovo, Chita, Ulan-Ude), N = 502; the Far East of Russia (Khabarovsk, Vladivostok, Petropavlovsk-Kamchatsky), N = 461; Central Russia (mostly Moscow and the Moscow Region), N = 157; and Ukraine (Rovno, Khmelnitsky, Ternopil, and other cities), N = 117. 

The Schwartz Value Survey (SVS) and Portrait Values Questionnaire (PVQ), in Russian by Karandashev [17], were used in this study. The use of two questionnaires made it possible to compare the intensity of all the ten types of values on each of the two scales or levels. The SVS reveals the personal values on the level of normative ideals, while the PVQ helps to study the values on the behavioral priorities level [17,18]. The study was performed in groups, anonymously, by a trained assistant. The obtained primary data were standardized to smooth down the differences in the scales ranges. For this purpose, generic R 3.5.1 function scale () was used with the default arguments: scale(x, center = TRUE, scale = TRUE). Each variable (column) was centered and then divided by the corresponding standard deviation. Ipsatization was not performed.

**Figure 1:**
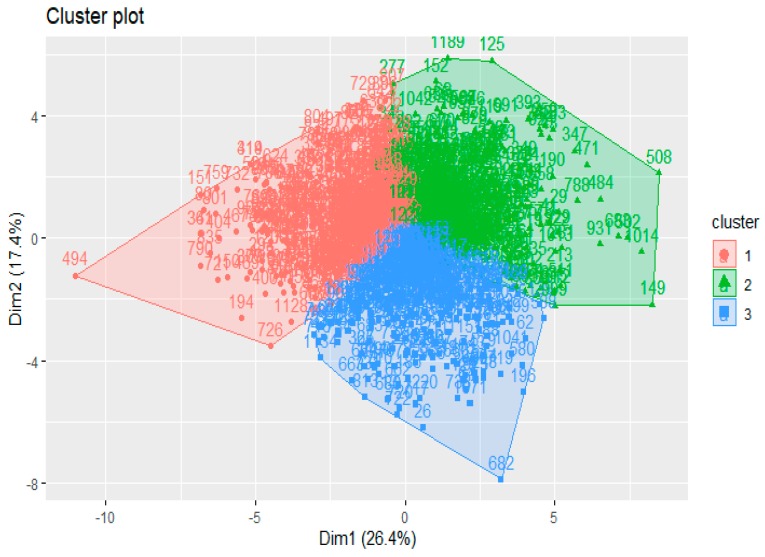
Ordinal diagram of the distribution of variables obtained from the Schwartz Value Survey (SVS) and Portrait Values Questionnaire (PVQ) projection on two principal components, three cluster solution.

The sample was divided into subgroups using the K-means cluster analysis in the R 3.5.1. statistical environment. As seen in Figure 1 there was a continuous multivariate distribution. We understood that any split in this case would be conditional. However, we believe that using this method is acceptable for identifying relatively homogeneous sub-samples of persons with particular value profiles. The NbClust 3.0 packet was used to determine the optimal number of clusters. A graphical method, Hubert Index was used to determine the number of clusters. The preferred variant was to split into three clusters (11 criteria out of 26).

**Figure 2:**
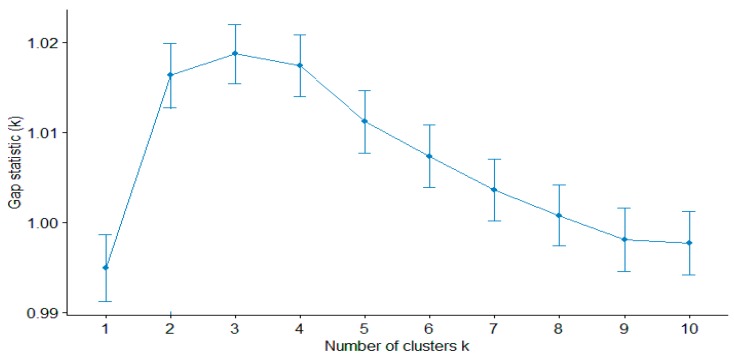
GAP statistics graph for selecting the optimal number of clusters.

Then we calculated the gap statistics, see Figure 2. The statistics for a three cluster solution had the greatest value. The statistics were calculated using the clusGup() function from the cluster package version: 2.1.0 in the R 3.5.1 statistical environment. The “firstmax” method was used to determine local maxima.

We used the Student *t*-test for linked samples for pair comparison, and the Pearson сhi-squared test for proportions comparison. For better visualization of data, a method of mosaic plots was used, showing the difference between the anticipated values, proceeding from the null hypothesis, and the observed values. 

## 3. Results

### 3.1. Schwartz Value Clusters

Using the method of cluster analysis, the whole sample of students was divided into three subgroups, or value clusters, each of them being rather homogenous under the following three aspects: (1) a pattern of values hierarchy, (2) the intensity of certain values expression, and (3) a certain proportion of values expression on two different scales or levels, measured separately by the SVS and PVQ, referred to as the normative ideals (NI) and the behavioral priorities (BP) scales.

**Figure 3:**
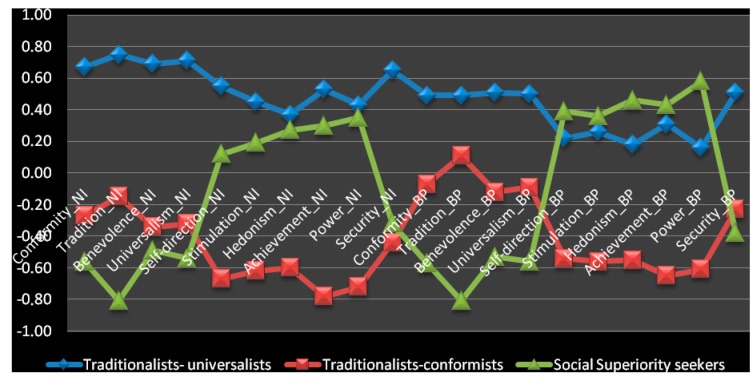
Schwartz Value Clusters in students (n = 1237). The items 1–10 indicate the importance of the 10 personal values on the normative ideals (NI) scale measured by the SVS, while the items 11–20 show the rate of the same 10 values on the scale of behavioral priorities (BP) measured by the PVQ.

Figure 3 represents in graphical form the three value clusters in the aspect of personal values hierarchy on both the normative ideals (NI) scale (values numbered from 1 to 10) and the behavioral priorities (BP) scale (values numbered from 11 to 20). In order to facilitate the juxtaposition of two scales in one, the data of the SVS and PVQ were standardized to make it possible to put them at single plotting scale.

In the hierarchical values structure in students of the first group, the most significant were *tradition, universalism, benevolence* and *conformity*, and the least significant of all, *hedonism* and *power*. The major goals of this group are respect and maintenance of tradition; understanding and tolerance; preserving and enhancing people’s welfare (mostly of loved ones); group solidarity; restraint of activities which may cause harm to anyone; safety, harmony, and stability of society, of relationships, and of oneself. Comparing the data obtained by the SVS and PVQ, one may suggest that on the BP scale (i.e., in the real behavioral pattern), compared to the NI scale, the intensity of all 10 values in this subgroup tends to lower under social pressure (Figure 3, Table 1). The *security* value lowers to the least extent so that on the behavioral level it remains among the respondents’ value priorities. Their values priority structure takes a plateau shape, all the five values *security, benevolence, universalism, tradition,* and *conformity* becoming almost equal in height, their significance levels ranging from 0.49 to 0.151. Together with the major value priorities, other personal values also tend to go down. The values *self-direction* and *power* are the first to decline in the real behavior (BP scale) as compared to the normative ideals. Along with a high need for security, stability, and predictability of the world, the respondents of this subgroup displayed a lower need for independent activity and personal success, with the second level (BP scale) still lower than on the first one (NI scale). Proceeding from the two most prominent values, together with their ratio on two scales, NI and BP, we denoted this group as *Traditionalists–Universalists* (*TrU*). It included 37 percent of the sample.

For the students belonging to the second group, all the 10 types of basic values proved to be of less importance as compared with the first group (Figure 3, Table 1). Although the values of utmost priority are the same—namely, *tradition, conformity, universalism,* and *benevolence*—the hierarchy inside the four prior values slightly differs. The value of *conformity* was ranked 2 instead of number 4 in the TrU group. The students of this group were oriented towards preserving stability through free will self-restraint and submission. They were concerned with the well-being of others, and not solely with their own affairs. Unlike the subjects from the TrU group, their value priorities were displayed more intensely on the BP scale than on the NI scale. It means that the expression of their value priorities was enhanced by the social impact, which might be due to their need to harmoniously interact with other people, a trait relevant to the value of *conformity*. The ratio of the *tradition* value rose more substantially on the behavioral level (BP scale). Proceeding from the two most prominent values, together with their ratio on the NI and BP scales, we denoted this group as *Traditionalists–Conformists* (*TrC*), and they comprised 35 percent of the sample. Like the *Traditionalists–Universalists* group, they were strongly oriented towards group interests and maintaining traditions. The main difference was that in the subjects belonging to the first (*TrU*) group, the values were more vividly expressed on the ideal level (NI scale), while the subjects of the second (*TrC*) group tended to display their major values more vividly in their actual behavior. It looks like the social impact tends to lower the idealism of the first group and reinforces the value priorities of the second. 

The third group looks like an antipode of both of the other groups. For the subjects of this group, the most significant values are *power, achievement,* and *hedonism*, an average significance is attached to *stimulation* and *self-direction*, while the denied values are *tradition, conformity, universalism, benevolence,* and *security*. Even more, in the actual behavior under the social impact, which is measured by the scale of behavioral priorities, the first-range values acquired still higher rates of expression (see Figure 3 and Table 1). In this group, the major driving motives were social superiority, personal success according to generally accepted standards, dominance upon people and resources, and acting in accordance with one’s own opinions. We denoted this group as *Social Superiority Seekers* (*SuS*)*,* and it comprised 28 percent of the sample. 

Now, we see that the studied sample was not uniform under the aspect of values structure and priorities. It broke into three clusters, *Traditionalists–Universalists* (*TrU*), *Traditionalists–Conformists* (*TrC*), and *Social Superiority Seekers* (*SuS*). 

### 3.2. Occurrence of Schwartz Value Clusters in Male and Female Subjects, Different Ethnic Groups, and Different Regions

The proposed typology was used as a basis for the second phase of the empirical study, as it helped us to examine the distribution of value types depending on a variety of socio-demographic factors. We found it useful to examine the rate of occurrence of the three value clusters in male and female students, in representatives of different ethnic communities, and in students coming from different geographic regions present the studied sample.

#### 3.2.1. Gender 

The used mathematical technique failed to reveal any significant difference in the occurrence of three value clusters among male and female subjects. It means that this type of cluster division, in the studied sample, had no gender diversity.

#### 3.2.2. Ethnicity

**Figure 4:**
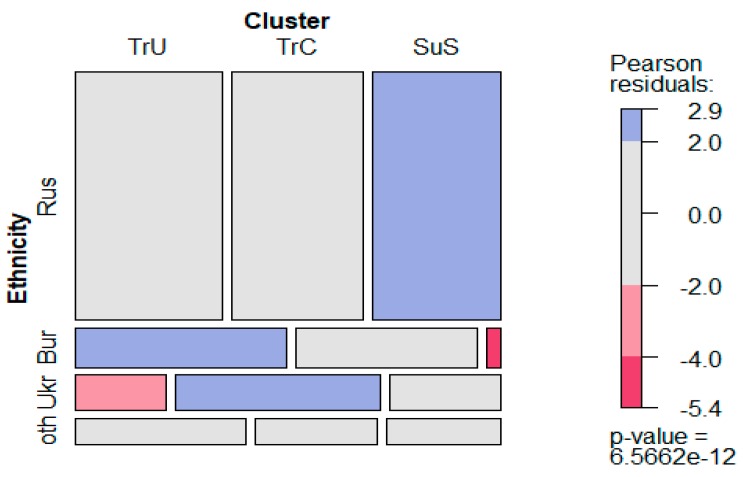
Occurrence of value clusters in different ethnic groups. Cluster abbreviations: TrU—Traditionalist–Universalist type, TrC—Traditionalist–Conformist type, SuS—Social Superiority Seekers type. Ethnicity abbreviations: Rus—Russian students, Bur—Buryat students, Ukr—Ukrainian students, oth—other ethnic groups.

The sample included students representing a variety of ethnic groups. The incidence of the value clusters was measured in representatives of three ethnic groups which were most numerous in the studied sample, namely in Russians, Buryats, and Ukrainians (Figure 4). Some other ethnic groups included only a few subjects each (total N = 93), not enough for statistical study, so we chose to treat them as an undifferentiated group, “Others” (Oth). The *Social Superiority Seekers* type (SuS) had a much higher incidence in Russian students (Rus) and significantly lower in Buryats (Bur). The *Traditionalists–Universalists* type (TrU) had a higher incidence in Buryats (Bur) and lower in Ukrainians (Ukr). *Traditionalists–Conformists* (TrC) were more common among Ukrainian students. 

#### 3.2.3. Geographic Region

**Figure 5:**
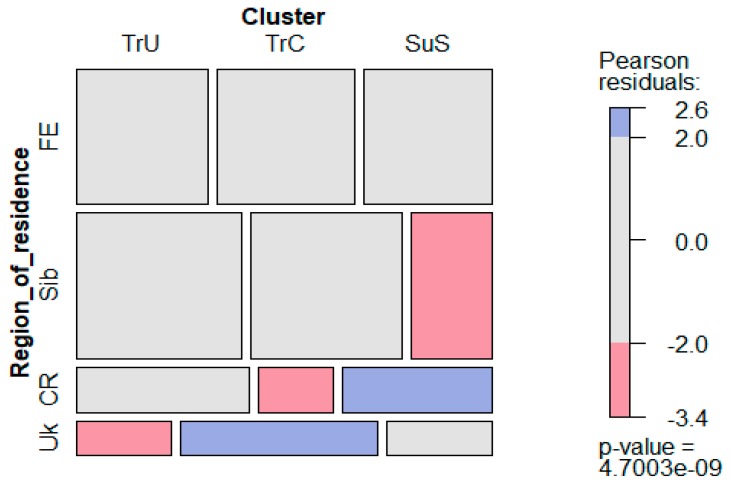
Occurrence of value clusters in different geographical regions. Region of residence abbreviations: FE—Far East of Russia, Sib—Siberian region, CR—Central Russia, Uk—Ukraine.

We considered the occurrence of value clusters in a number of geographic regions (Figure 5). The results are as follows. The *Social Superiority Seekers* (SuS) type had a significantly rarer incidence in the Siberian region (Sib) and was significantly more often met in the region of Central Russia (CR). The *Traditionalists–Universalists* (TrC) type was less often met in Ukraine (Ukr), while the *Traditionalists–Conformists* (TrC) type had a higher incidence in Ukraine and a lower incidence in Central Russia (CR).

## 4. Discussion

The obtained results disclosed a certain heterogeneity of value priorities in the studied sample of present-day university students. It had a definite polarization along two virtual axes or pairs of oppositions. The first pair is *social focus vs personal focus*, and the second one refers to acceptance of one of two opposite groups of values - *values of conservation* (*tradition*, *conformity*, and *security*) ***vs*** *values of openness to change* (*stimulation*, *self-direction* and, partly, *hedonism*) (grouping by Schwartz [3,4]). *Traditionalists–Universalists* and *Traditionalists–Conformists*, despite being different in the rate of values expression on the level of ideals (the NI scale) and behavioral level (the BP scale), both had a fundamentally common hierarchy of values, with the top four *tradition, universalism*, *benevolence* and *conformity*. Both types were oriented towards the social focus, towards the value groups of self-transcendence and сonservation. They attributed great importance to the common welfare, they were apt to yield their own interests to the goals of the community, and they tended to prefer a collective action. They constituted together 72 percent of the sample.

Our findings have much in common with the study performed by N. M. Lebedeva and A. N. Tatarko [19], dealing with the value priorities of Russian people on the individual level. In addition to universal motivational patterns, the authors discovered one more specific motivational pattern which combines the values *benevolence*, *universalism,* and *security;* they describe it as *‘universal responsiveness’.* The authors appeal to a very special type of individual motivation immanent to Russian cultural code. “For the sake of the common good,...one should try all ways to meet other people’s needs, to restrict and confine oneself, which makes good for all, both your neighbors, and relatives, and strangers; in another words, the harmony and well-being of family and society is based on our personal benevolence and self-restriction” ([19], p. 74). In accordance with this interpretation of the leading type of value priorities, we should also mention that in the present study, the prevalent value pattern included, among others, *tradition* and *conformity*. It may be assumed that the presence of these values in our respondents’ world outlook also serves them as a means of acquiring a sense of well-being and stability of community, family, and the world on the whole.

On the other hand, there is a subgroup of *Social Superiority Seekers* whose value structure was contradictory to the above-described type. These subjects were chiefly oriented towards themselves and the interests of their own; they were focused on leadership, superiority, and individual achievement, while the value of *conformity* was of little importance for them. Instead, they were ambitious and open to changes. This group was 28 percent of the sample. 

The high percentage of *traditionalists* in the sample of young people might be due to the socio-political factors in the current Russian society, where all kinds of traditional values are strongly supported and reinforced. The values clusters were irrespective of gender but showed a variation in respondents depending on their site of residence and ethnic identity, which also suggests an important role of the socio-cultural environment in formation of a personal values system. 

The rank-based analysis of value hierarchies allowed us to claim congruence between the values clusters established by Lee and her colleagues [6] and by ourselves. The value hierarchies of the *Traditionalists–Universalists* and *Traditionalists–Conformists* types in the present study resemble the hierarchies of the in-group clusters of Lee’s American sample, the main priorities being *benevolence*, *conformity*, and *traditions* [6]. The difference is in the fact that in the *Traditionalists–Universalists* and *Traditionalists–Conformists*, among the most significant values there is *universalism,* while in the description of Lee’s cluster in-group one may encounter the value of *self-direction*. In the Chinese sample, in the structure of the in-group cluster, the *tradition* value displays less significance compared to the American sample (rank 10 vs 3) [6]. The *Social Superiority Seekers* cluster, in the present study, resembles the *self-enhancement* cluster from the cited paper, where the first positions were *power, achievement, hedonism, stimulation,* and *self-direction*. 

The above-cited features suggest that not only the values themselves are universal for different countries and nations, but the specific personal values patterns or types might prove universal as well, which will demand further studies. Such types as *traditionalists* or *social superiority seekers* may come out to be universal value types in every kind of society, though their quantitative relations may vary to a greater or lesser extent, depending on socio-cultural conditions existing in different nations and historical periods. 

The results of this study may extend our capabilities for the prediction of the social, economic, and political behavior of the younger generation. 

## Figures and Tables

**Table 1:** Cluster Descriptive Statistics.

	**Traditionalists–Universalists (37%)**			**Traditionalists–Conformists (35%)**			**Social Superiority Seekers (28%)**		
**Normative Ideals**	**Rank**	***M***	***SD***	**Rank**	***M***	***SD***	**Rank**	***M***	***SD***
Conformity	4	**0.67** ***	0.72	2	*−0.27*	0.93	9	*−0.56*	0.9
Tradition	1	**0.75** ***	0.76	1	*−0.15*	0.8	10	*−0.81*	0.78
Benevolence	3	**0.69** ***	0.7	4	*−0.34*	0.93	7	*−0.49*	0.92
Universalism	2	**0.71** ***	0.76	3	*−0.32*	0.89	8	*−0.54*	0.86
Self-direction	6	**0.55** ***	0.74	8	*−0.67*	0.94	5	**0.12**	0.87
Stimulation	8	**0.45** ***	0.87	7	*−0.62*	0.89	4	**0.19**	0.88
Hedonism	10	**0.37** ***	0.82	6	*−0.6*	0.95	3	**0.27**	0.93
Achievement	7	**0.53** ***	0.74	10	*−0.78*	0.89	2	**0.3**	0.8
Power	9	**0.43** ***	0.89	9	*−0.7* *	0.79	1	**0.35**	0.85
Security	5	**0.65** ***	0.76	5	*−0.44*	0.95	6	*−0.32*	0.89
	**Traditionalists–Universalists (37%)**			**Traditionalists–Conformists (35%)**			**Social Superiority Seekers (28%)**		
**Behavioral Priorities**	**Rank**	***M***	***SD***	**Rank**	***M***	***SD***	**Rank**	***M***	***SD***
Conformity	4.5	**0.49**	0.86	2	−0.07 ***	0.96	9	*−0.57*	0.91
Tradition	4.5	**0.49**	0.9	1	**0.11** **	0.88	10	*−0.81*	0.75
Benevolence	1.5	**0.51**	0.91	4	*−0.12* ***	0.88	7	*−0.53*	0.94
Universalism	3	**0.5**	0.89	3	−0.09 ***	0.9	8	*−0.56*	0.93
Self-direction	8	**0.22**	0.94	6	*−0.54* *	0.99	4	**0.39** ***	0.77
Stimulation	7	**0.26**	0.95	8	*−0.56*	0.92	5	**0.36** ***	0.84
Hedonism	9	**0.18**	0.92	7	*−0.55*	0.97	2	**0.46** **	0.81
Achievement	6	**0.31**	0.89	10	*−0.65* *	0.89	3	**0.43** *	0.83
Power	10	**0.16**	0.98	9	*−0.61* *	0.76	1	**0.58** ***	0.86
Security	1.5	**0.51**	0.89	5	*−0.23* ***	0.94	6	*−0.38*	0.93

Note: Cluster values printed in bold were significantly higher than the total sample, and those printed in italics were significantly lower at the 95% level. Cluster values marked asterisks * *p* < 0.05; ** *p <* 0.01; *** *p <* 0.001) were significantly higher than their counterpart on the other scale in the same cluster (NI compared to BP).
